# Laparoscopy in the management of pediatric vesicoureteral reflux

**DOI:** 10.4103/0970-1591.36716

**Published:** 2007

**Authors:** Atul A. Thakre, B. Sreedhar, C. K. Yeung

**Affiliations:** Division of Pediatric Surgery and Pediatric Urology, Department of Surgery, Prince of Wales Hospital, Chinese University of Hong Kong, Hong Kong SAR, China

**Keywords:** CO_2_ pneumovesicum, da Vinci robot, laparoscopy, MIS, ureteric reimplantation, vesicoureteral reflux

## Abstract

The prevalence of vesicoureteral reflux (VUR) has been estimated as. 4 to 1.8% among the pediatric population. In children with urinary tract infection the prevalence is typically from 30-50% with higher incidence occurring in infancy. When correction of VUR is determined to be necessary, traditionally open ureteral reimplantation by a variety of techniques has been the mainstay of treatment. This approach is justified because surgical correction affords a very high success rate of 99% in experienced hands and a low complication rate. In that context the purpose of this review article is to highlight the use of laparoscopy and robot-assisted techniques to perform ureteric reimplantation for the management of pediatric VUR. A detailed review of recent literature on the subject is performed to find out various aspects of minimally invasive surgery in the treatment of VUR, highlighting evolution of management approaches, operative steps, complications, results and the current status in clinical practice. We also share our experience on the subject.

Vesicoureteral reflux (VUR), the most common urological abnormality in children, is considered as a significant factor for the development of urinary tract infection (UTI), progressive renal damage or scarring and end stage renal failure.[1–6] Surgical management of vesicoureteral reflux involves ureteral reimplantation and endoscopic subureteric injection of bulking agent. Subureteral injection of implant materials has shown much promise in recent years, with success rates approaching open surgery after two or more injections.[7] However, surgical treatment for VUR by open ureteral reimplantation has remained the gold standard of surgical intervention.[7–9] Despite excellent long-term results of various open reimplantation techniques the commonly performed intravesical approach involves in-splitting of the abdominal wall, forced retraction of the bladder and long postoperative urinary diversion causing pain, bladder spasms and longer hospital stay.[10] Even the open extravesical technique for ureteral reimplantation, has had the drawback of postoperative urinary retention, especially in bilateral ureteric reimplantation.[10]

In the last decade there has been a shift to perform ureteric reimplantation using the laparoscopic approach in order to provide all advantages of a minimally invasive technique and long-term results similar to traditional open ureteric reimplantation.

## EVOLUTION OF LAPAROSCOPIC APPROACH FOR URETERIC REIMPLANTATION

The first laparoscopic extravesical (transperitoneal) approach was used to perform modified Lich-Gregoir reimplantation. The Lich-Gregoir method, which evolved in the 1960s, is the most popular extravesical procedure and the first laparoscopic extravesical approach was performed by using the modified Lich-Gregoir technique. In 1993 and 1994, several groups reported experimental efforts with extravesical laparoscopic reimplantation in the porcine model 4,5 where animals first had reflux created by endoscopically incising the submucosal tunnel. Using a transperitoneal approach, on the posterior wall of the bladder a detrusor incision was created proximal to the ureteral hiatus, the ureter was positioned within the trough and the detrusor closed over it with either staples or absorbable sutures. The early results were promising although investigators reported it to be technically challenging. Shortly after the initial reports of extravesical ureteral reimplantation, experience with a combined transvesical and transurethral endoscopic approach to ureteral reimplantation was reported by two groups.[11,12] In 1996, Cartwright *et al*., reported their experience in 22 children who underwent percutaneous endoscopic trigonoplasty.[12] Reflux was of moderate grade in most cases and resolved in 20 of 32 ureters at six months, for a resolution rate of 62.5%. Gill and associates in 2001 reported their series utilizing the laparoscopic technique described by Cartwright and associates. Of three patients, reflux resolved in two and persisted as Grade II in one patient at six-month follow-up.[13]

Following this initial disappointing resolution rate with trigonoplasty, members of the same group performed transvesical mobilization of the ureters again using two suprapubic transvesical ports and a cystoscope.[14] Using similar instrumentation and a stent within the ureteral orifice, the ureteral orifices were dissected from their detrusor attachments using miniature laparoscopic scissors. Dissection gained approximately 2.5 cm of ureteral mobility into the intravesical space. No further mobilization was attempted owing to concern of rapid escape of CO_2_ into the retroperitoneal space and subsequent pneumoretroperitoneum and bladder collapse. The ureters were then reimplanted in a cross-trigonal fashion after the detrusor hiatus had been reapproximated with interrupted polyglactin sutures. The length of the submucosal tunnel was limited and the procedure probably achieved a 2 to 2.5 cm ureteral reimplantation on each side. At one year follow-up, 10 of 12 ureters managed in this fashion showed no reflux.

In an effort to improve on their results, Okamura and associates developed a new procedure called endoscopic trigonoplasty II.[15,16] This was based on the open antireflux procedure reported by Orikasa.[17] Using the same approach (cystoscope and trocar placement) as the endoscopic trigonoplasty I, this procedure created a U-shaped flap including the ureter, tightened the muscular backing and then elongated the intramural ureter. In their initial experience reflux resolution was observed in 86% at one year follow-up.

In 2003 laparoscopic Cohen procedure using a pneumovesical approach was first described in an animal model.[18] We developed this pilot animal model using piglets where we found that under carbon dioxide insufflation of the bladder at around 10 mmHg pressure, a large potential working space could be obtained that would allow various intravesical procedures, including a Cohen's type of cross-trigonal ureteral reimplantation, using standard laparoscopic instruments.[18]

## SURGICAL TECHNIQUES OF LAPAROSCOPIC URETERIC REIMPLANTATION

### Intravesical reimplantation

**Figure 1:**
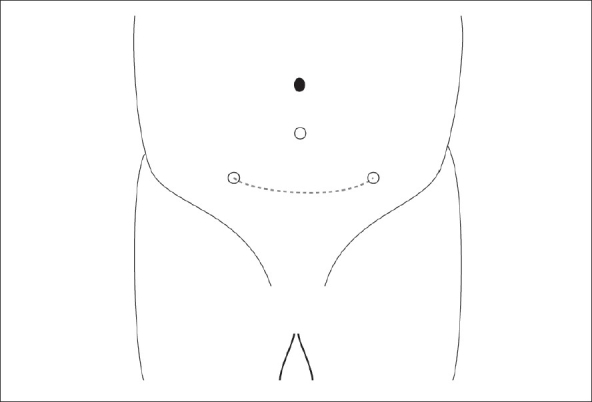
5 mm working ports inserted along the interspinous skin crease on either side of the lower lateral wall of the distended bladder under vesicoscopic guidance

**Figure 2:**
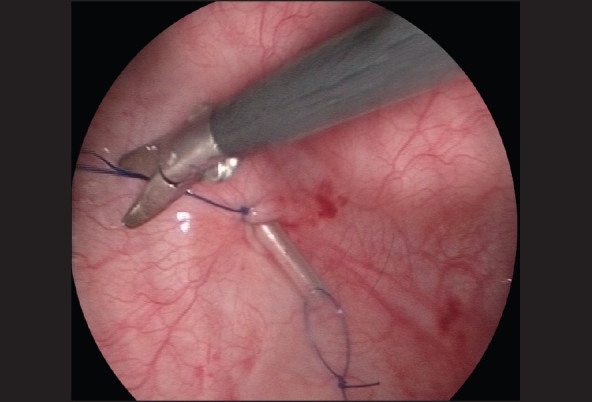
A 3–4 cm long segment of a Fr 4 or 5 catheter is inserted into the ureter as a stent to facilitate subsequent ureteral mobilization and dissection and secured with a 4-zero monofilament suture

The patient is positioned supine with the legs separated apart for cystoscopy and bladder catheterization intraoperatively. For small infants the surgeon can stand and operate over the patient's head whereas for older children, the surgeon usually stands on the patient's left side. The video column is placed between the patient's legs at the end of the table. The port placement is preceded by transurethral cystoscopy to allow placement of the first camera port under cystoscopic guidance. The bladder is first distended with saline and a 2-0 monofilament traction suture is passed percutaneously at the bladder dome under cystoscopic vision, through both the abdominal and bladder walls. This helps to keep the bladder wall from falling away when the first camera port site incision is made and during insertion of the cannula. A 5-mm Step port (Inner Dyne Inc, USA) is then inserted under cystoscopic vision. A urethral catheter is then inserted to drain the bladder and start carbon dioxide insufflation to 10-12mmHg pressure. The urethral catheter is used to occlude the internal urethral meatus to secure CO_2_ pneumovesicum and it could also serve as an additional suction irrigation device during subsequent dissection and ureteric reimplantation. A 5-mm 30-degree scope is used to provide intravesical vision. Two more 3-5 mm working ports are then inserted along the interspinous skin crease on either side of the lower lateral wall of the distended bladder under vesicoscopic guidance [Figure 1]. A 3–4 cm long segment of a Fr 4 or 6 catheter is then inserted into the respective ureter as a stent to facilitate subsequent ureteral mobilization and dissection and secured with a 4-zero monofilament suture [Figure 2]. Intravesical mobilization of the ureter, dissection of submucosal tunnel and a Cohen's type of ureteral reimplantation is then performed under endoscopic guidance, in a similar manner to the open procedure.

**Figure 3:**
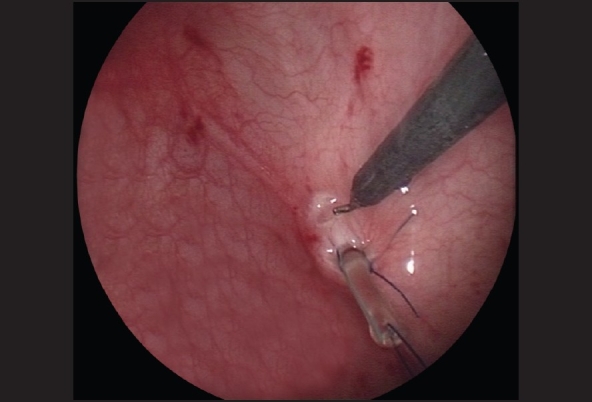
The ureter is mobilized by first circumscribing it around the ureteral orifice using hook electrocautery

**Figure 4:**
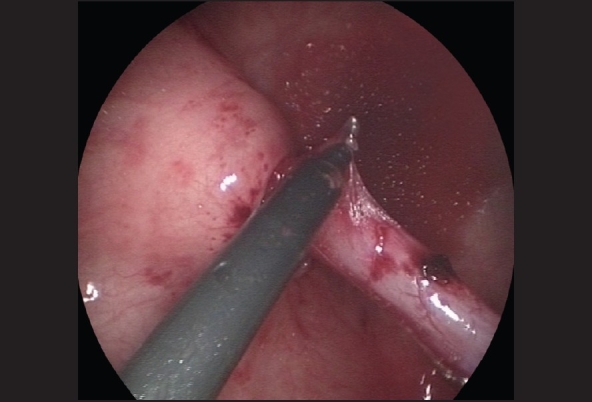
With traction on the ureteric stent using a blunt grasper, the fibrovascular tissue surrounding the lower ureter can be seen and divided using fine 3-mm endoscopic scissors and diathermy hook, while preserving the main ureteric blood supply

**Figure 5:**
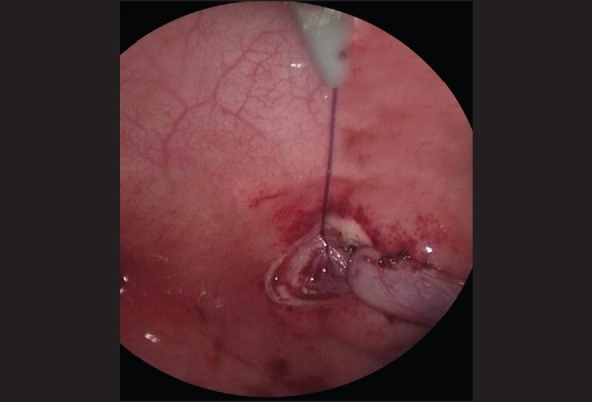
Once adequate ureteral length is obtained, the muscular defect in the ureteral hiatus is repaired using 5-zero absorbable sutures, usually with an extracorporeal knot tying technique

**Figure 6:**
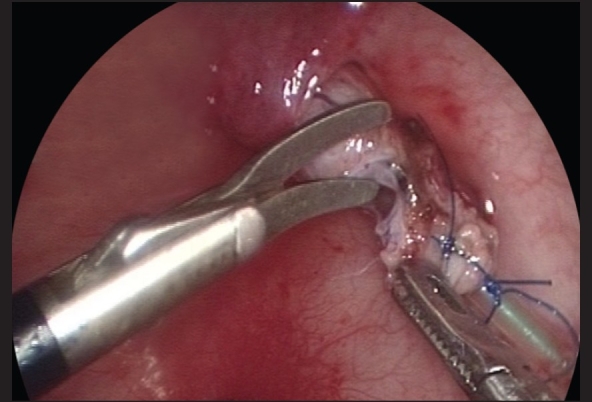
Ureteroneocystostomy is performed under endoscopic guidance with intracorporeal suturing using interrupted 5–0 or 6–0 poliglecaprone or polydioxanone sutures

**Figure 7:**
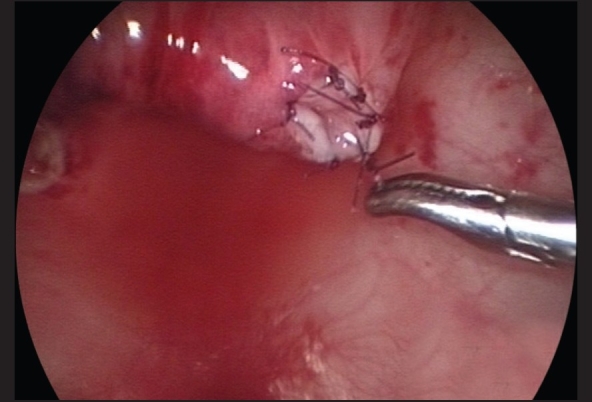
Completed ureteroneoctostomy

The ureter is mobilized by first circumscribing it around the ureteral orifice using hook electrocautery [Figure 3]. With traction on the ureteric stent using a blunt grasper, the fibrovascular tissue surrounding the lower ureter can be seen and divided using fine 3-mm endoscopic scissors and diathermy hook, while preserving the main ureteric blood supply [Figure 4]. Mobilization of the ureter is continued for 2.5 to 3 cm to the extravesical space. Once adequate ureteral length is obtained, the muscular defect in the ureteral hiatus is repaired using 5-zero absorbable sutures, usually with an extracorporeal knot tying technique [Figure 5]. A submucosal tunnel is then created as in an open Cohen's procedure. Using a diathermy hook, a small incision is made over the future site of the new ureteral orifice, usually chosen to be just above the contralateral ureteral orifice. Dissection of the submucosal tunnel is then started from the medial aspect of the ipsilateral ureteral hiatus towards the new ureteral orifice, using a combination of endoscopic scissor dissection and diathermy hook for hemostasis. Once the submucosal tunnel dissection is completed, a fine grasper is passed and the mobilized ureter is gently drawn through the tunnel. Ureteroneocystostomy is performed under endoscopic guidance with intracorporeal suturing using interrupted 5–0 or 6–0 poliglecaprone or polydioxanone sutures [Figures 6–7]. A ureteral stent is not routinely used except for selected patients undergoing bilateral ureteral reimplantation or those with megaureters requiring tapering ureteroplasty. The working ports are removed under endoscopic vision with evacuation of the pneumovesicum. The bladder-holding stitches are then tied. Each port site entry wound is then closed with a subcuticular monocryl suture. The bladder catheter is kept in place for one day and the patient is discharged home and advised to refrain from play for a few days.

We have not faced any major complications with this technique. In the early part of the series when the cannulas were not secured to the bladder wall, displacement of the port outside the bladder wall occurred. This resulted in gas leakage into the extravesical space, with compromise of the intravesical space and endoscopic vision. It is usually possible to reintroduce the ports but securing the ports perfectly is the key to the success of this technique.[10] We have experienced mild to moderate scrotal and suprapubic emphysema immediately postoperatively, which subsided spontaneously within 24h. Persistent mild hematuria up to 72h has also been observed, which too settles spontaneously. A recent series has reported complications of postoperative urinary leak in 12.5% and ureteral stricture at the neoureterovesical anastomosis in 6.3%. This series also reported higher complications in patients two years or younger with bladder capacity less than 130 cc.[19]

Our operative success for laparoscopic Cohen performed in 60 children (46 girls and 14 boys) with an average age 1.81 years (range one month-7.24 years) and a mean follow-up of two years (range 1.1–5 years) has been 97.6%. These results are encouraging and endoscopic intravesical ureteric mobilization and cross-trigonal ureteral reimplantation can be safely performed with routine pediatric laparoscopic surgical techniques and instruments under carbon dioxide insufflation of the bladder.[10,19]

### Extravesical reimplantation

The extravesical approach can be performed unilaterally or bilaterally, applying Lich-Gregoir technique. The patient is in supine position for an extravesical laparoscopic ureteral reimplantation. The bladder is first inspected with cystoscopy. A 3-Fr ureteral catheter may be placed at this time to help laparoscopic identification although we do not routinely use it. After scopy the bladder is drained by a catheter. An infraumbilical incision is made to place a 5-mm trocar for vision by open method. The other two trocars are placed along the lateral border of the rectus. Ports are fixed to the abdominal wall using a stitch which is also used to close the fascia. The ureter is normally seen well at the pelvic brim and can be followed to its insertion into the bladder. The technique follows the same steps as the open Lich-Gregoir procedure. It starts by dissecting the ureter after opening the peritoneum just over the posterior bladder wall. In females the ureter can be found in the anterior relation to the uterus. The ureter is freed from the surrounding tissue keeping its vessels intact. Approximately 4-5 cm is dissected to permit mobility and to prevent kinking as the bladder tunnel is created for the ureter. It is important to take care not to damage the Vas during the dissection around the UV junction. Adequate exposure of the posterior bladder wall is a key factor in this operation. A hitch stitch through the posterior bladder wall can be used to improve the exposure of the ureteral hiatus, attaching to the abdominal wall or through it.

Once the ureter is free, the size of the tunnel is estimated after the bladder is partially distended. The ureter should course slightly laterally to avoid kinking of the ureter. A tunnel is adequately dissected to obtain a 5:1 ratio of length to width; the detrusor muscle is divided full thickness using a cautery hook while keeping the mucosa intact. Any perforations of the mucosa are closed using a 6-0 chromic suture. The ureter is positioned in the tunnel so as to avoid any kinking or excessive compression of the ureter to prevent obstruction. Closure may be from proximal end of the incision to distal or in the reverse fashion. In the latter, the ureter is well visualized while in the former needle needs to be passed under the ureter each time. The peritoneum is closed in a running fashion and a catheter is left in place, for one day. The indications for using the MIS extravesical Lich-Gregor approach are the same as for the open surgical technique although some investigators do not find the extravesical procedure appropriate for patients with megaureters requiring tapering.[9]

The common complication is accidental bladder mucosal perforations during the dissection of the detrusor muscle trough for the submucosal tunnel. The bladder mucosa perforations can be prevented by not over distending the bladder and use of blunt instruments like the suction tip to do the dissection of the mucosa from the detrusor muscle. Another limitation of this approach is transgressing the peritoneal cavity and difficult retraction of the bladder for the want of good exposure.

Lakshmanan and Fung reported extravesical laparoscopic reimplant in 99 ureters in 66 children aged four to 18 years.[9] With the first 13 cases (the initial learning curve) of their series excluded, they reported a success rate of 97.8% with a mean follow-up of 34 months. Shu *et al*., in 2004 have taken a similar approach and have documented excellent results and recovery in the post pubertal patients.[20] Peters and associates in 2005 reported 90% results for their extravesical approach by robot-assisted laparoscopy.[21] They also highlighted the crucial advantage of avoiding injury to the ureter and the urothelium by the transperitoneal extravesical approach.

## ROBOT-ASSISTED LAPAROSCOPY

This technology has provided an opportunity to apply new techniques to practice MIS. From our own experience we believe that one of the main advantages of the robotic system, especially in children, is of offering surgeons inexperienced in laparoscopy the benefits of high dexterity in minimally invasive procedures and for the experienced laparoscopic surgeon to improve their capacity.[22] Robot -assisted laparoscopy has complemented and helped to overcome some of the limitations of conventional laparoscopic techniques, particularly for pediatric reconstructive surgeries.[23,24] The da Vinci surgical system, provides the advantage of three-dimensional visualization with tremor-filtered instrument movement and articulating instruments with six degrees of freedom to do delicate manipulation for reconstructive surgery.[25] Applications of this technology have evolved and are now achieving identical results as in open surgery and using suture as small as used in open surgery, 6-0 or 7-0.

The robot-assisted extravesical approach for treating VUR can be performed unilaterally or bilaterally, following the same steps as have been described in the laparoscopic section. Typically, the patient is in the supine position and legs placed apart. An open technique is used to place the first trocar, the 12mm camera port, in the umbilicus. The working ports, 8mm, are positioned in the mid-clavicular line bilaterally, about 1cm below the umbilical line. Ports are fixed and secured firmly to the abdominal wall using a stitch which is also used to close the fascia later. The robot is docked over the child's feet end to perform the surgery. For the robotic Cohen procedure the patient and robot docking position is the same as described for the extravesical approach. The procedure steps are the same as described earlier in the laparoscopic pneumovesicum reimplantation technique. While doing a robot-assisted procedure the main difference is the use of 12mm camera port and two 8mm working ports. Our experience of conventional lap for this technique has been quite different. The working space after all the three robotic ports have been placed inside the bladder is quite limiting as against what we usually experience with 5mm ports and 3mm instruments doing this surgery. There isn't enough data doing this technique by using robotic assistance.[26] In our experience robot-assisted reimplant approaches both intravesically and extravesically but we suspect that the greatest benefit may lie with extravesical approach. The technique remains challenging using the robotic system and early results are not uniformly successful. It is difficult to accurately assess the impact on reduction of morbidity, but in all, the enhanced visualization and dexterity are noticeable.

Disadvantages of this robotic system include the lack of tactile sensation and thus visualization of anatomic landmarks is the key to successfully completing the operation. The unscrubbed surgeon is away from the operating table and therefore must depend on an experienced scrubbed assistant. Active communication between the primary surgeon, first assistant and staff is imperative. Although the learning curve for the surgeon may be short there is a substantial learning curve for the ancillary staff. Finally, the cost of the da Vinci robot is always a consideration. An initial investment of over $1,000,000 and subsequent running costs of $80,000 to $100,000 a year may not make this procedure feasible at many centers.

Robotic surgery in pediatric urology is an evolving technique. The computer-assisted system has introduced smaller instruments (5mm) which are available although do not provide any added advantage in efficiency, primarily due to their design and the monocular lens system. Another improvement in design is the addition of a fourth arm which can be applied as a retractor and developing a smaller robot with better maneuverability while docking. As the technology continues to get better, the efficiency of the robotic system is likely to improve and offer the means to overcome impediment in children. Animal studies demonstrate robotic assistance can increase the applicability of minimally invasive surgery to complex procedures in children and neonates;[27] however, the ultimate role of robotic-assisted or computer-assisted surgical systems remains unclear.

To further determine the role of robotics in performing ureteric reimplantation in children, rigorous prospective research is needed that combines surgical and technical outcomes with overall subjective or cosmetic outcome and economic analysis.[28]

## DISCUSSION

Laparoscopic surgery has gradually made its place in surgically dealing VUR. Laparoscopic and robot-assisted extravesical and intravesical surgeries have shown good early results.[10,19,21] It has also been shown that children benefit from the improved cosmesis, more rapid recovery and decreased postoperative analgesia requirements with the laparoscopic technique. Initial experience reported increased operative time; however, this has been overcome with greater experience.[10,19] Ureteric reimplant surgery for vesicoureteric reflux offers consistent long-term cure, i.e. complete resolution of reflux, no VUR recurrence and no ureteric obstruction. Performing ureteric reimplantation using minimally invasive technique makes anti-reflux surgery more acceptable as it takes away most of the disadvantages of open anti-reflux surgeries. Our experience demonstrated that greatest technical merit with high level of surgical precision is required to perform this surgery. The operation requires extreme care, gentleness and tissue respect while dissecting out the ureters. Great care needs to be taken to prevent damage to the ureteric vascularity which is an important cause which leads to developing ureteric necrosis and strictures. This surgery needs to have a very good submucosal tunnel of sufficient length and width to accommodate the refluxing ureter. Laparoscopy aids fine dissection of the ureter and the submucosal tunnel with minimal trauma to the bladder wall and mucosa. The bladder can be quickly rehabilitated after surgery and normal voiding ensues in the long term. No long-term voiding dysfunction has been noted. To obtain the highest possible success with these operations the decisive technical details described should be meticulously observed.[10] Thus from our experience the technique described is a good alternative to the standard intravesical or extravesical techniques to correct primary VUR. Although, the technique definitely mandates very good laparoscopic reconstruction skills to achieve these desired results.

The future of transvesical and extravesical minimally invasive procedures in children is promising and should be most interesting to follow over the next five to 10 years. Progress in the area of transvesical and extravesical surgery for treating vesicoureteral reflux in children will be made as more and more surgeons take up these procedures by laparoscopy and also by robot-assisted procedure with future innovations in robotic instruments.

## References

[1] Bailey RR, Maling TM, Swanison CP, Schrier RW, Gottschalk CW (1993). Vesicoureteral reflux and reflux nephropathy. Diseases of the Kidney.

[2] Weiss R, Duckett J, Spitzer A (1992). Results of a randomized clinical trial of medical versus surgical management of infants and children with grade 3 and 4 primary vesicoureteral reflux (United States) The international Reflux Study in Children. J Urol.

[3] Lich R, Howerton LW, Davis LA (1961). Vesicourethrography. J Urol.

[4] Atala A, Kavoussi LR, Goldstein DS, Retik AB, Peters CA (1993). Laparoscopic correction of vesicoureteral reflux. J Urol.

[5] Schimberg W, Wacksman J, Rudd R, Lewis AG, Sheldon CA (1994). Laparoscopic correction of vesicoureteral reflux in the pig. J Urol.

[6] McDougall EM, Urban DA, Kerbl K, Clayman RV, Fadden P, Royal HD (1995). Laparoscopic repair of vesicoureteral reflux utilizing the Lich-Gregoir technique in the pig model. J Urol.

[7] Lakshmanan Y, Fung LC (2000). Laparoscopic extravesicular ureteral reimplantation for vesicoureteral reflux: recent technical advances. J Endourol.

[8] Ehrlich RM, Gershman A, Fuchs S (1994). Laparoscopic vesicoureteroplasty in children: Initial case reports. Urology.

[9] Janetschek G, Radmayr C, Bartsch G (1995). Laparoscopic ureteral anti-reflux plasty reimplantation: First clinical experience. Ann Urol (Paris).

[10] Yeung CK, Sihoe JD, Borzi PA (2005). Endoscopic cross-trigonal ureteral reimplantation under carbon dioxide bladder insufflation: A novel technique. J Endourol.

[11] Okamura K, Ono Y, Yamada Y, ato T, Tsuji Y, Ohshima S (1995). Endoscopic trigonoplasty for primary vesicoureteric reflux. Br J Urol.

[12] Cartwright PC, Snow BW, Mansfield JC, Hamilton BD (1996). Percutaneous endoscopic trigonoplasty: A minimally invasive approach to correct vesicoureteric reflux. J Urol.

[13] Gill IS, Ponsky LE, Desai M, Kay R, Ross JH (2001). Laparoscopic cross-trigonal Cohen ureteroneocystostomy: Novel technique. J Urol.

[14] Gatti JM, Cartwright PC, Hamilton BD, Snow BW (1999). Percutaneous endoscopic trigonoplasty in children: Long-term outcomes and modifications in technique. J Endourol.

[15] Okamura K, Kato N, Takamura S, Tanaka J, Nagai T, Watanabe H (1997). Trigonal splitting is a major complication of endoscopic trigonoplasty at 1-year follow-up. J Urol.

[16] Tsuji Y, Okamura K, Nishimura T, Okamoto N, Kobayashi M, Kinukawa T (2003). A new endoscopic ureteral reimplantation for primary vesicoureteral reflux (endoscopic trignoplasty II). J Urol.

[17] Orikasa S (1990). A new antireflux procedure. Eur Urol.

[18] Olsen LH, Deding D, Yeung CK, Jorgensen TM (2003). Computer assisted laparoscopic pneumovesical ureter reimplantation a.m. Cohen: Initial experience in a pig model. APMIS Suppl.

[19] Kutikov A, Guzzo TJ, Canter DJ, Casale P (2006). Initial experience with laparoscopic transvesical ureteral reimplantation at the Children's Hospital of Philadelphia. J Urol.

[20] Shu T, Cisek LJ, Moore RG (2004). Laparoscopic extravesical reimplantation for postpubertal vesicoureteral reflux. J Endourol.

[21] Peters CA, Woo R (2005). Intravesical robotically assisted bilateral ureteral reimplantation. J Endourol.

[22] Peters CA (2004). Robotically assisted surgery in pediatric urology. Urol Clin North Am.

[23] Peters C (2003). Laparoscopy in paediatric urology: Adoption of innovative technology. BJU Int.

[24] Peters CA (2006). Robotic-assisted laparoscopy applied to reconstructive surgeries in children. World J Urol.

[25] Dasgupta P, Challacombe B (2004). Robotics in urology. BJU Int.

[26] Peters CA, Woo R (2005). Intravesical robotically assisted bilateral ureteral reimplantation. J Endourol.

[27] Lorincz A, Knight CG, Kant AJ, Langenburg SE, Rabah R, Gidell K (2005). Totally minimally invasive robot-assisted unstented pyeloplasty using the Zeus Microwrist Surgical System: An animal study. J Pediatr Surg.

[28] Lee RS, Broer JG (2006). Robotic surgery for ureteropelvic junction obstruction. Curr Opin Urol.

